# Quantitative MRI imaging of parenchyma and venation networks in *Brassica napus* leaves: effects of development and dehydration

**DOI:** 10.1186/s13007-024-01187-2

**Published:** 2024-05-13

**Authors:** Pierre-Nicolas Boulc’h, Guylaine Collewet, Baptiste Guillon, Stéphane Quellec, Laurent Leport, Maja Musse

**Affiliations:** 1 UR Optimisation des Procédés en Agro-alimentaire, Agriculture et Environnement (OPAALE), INRAE, 35000 Rennes, France; 2 UMR Institut de Génétique, Environnement et Protection des Plantes (IGEPP), INRAE, Institut Agro Rennes-Angers, Univ Rennes, 35653 Le Rheu, France

**Keywords:** Winter oilseed rape, Water relations, T_2_ mapping, Transverse relaxation time, Water stress, Dehydration

## Abstract

**Background:**

Characterisation of the structure and water status of leaf tissues is essential to the understanding of leaf hydraulic functioning under optimal and stressed conditions. Magnetic Resonance Imaging is unique in its capacity to access this information in a spatially resolved, non-invasive and non-destructive way. The purpose of this study was to develop an original approach based on transverse relaxation mapping by Magnetic Resonance Imaging for the detection of changes in water status and distribution at cell and tissue levels in *Brassica napus* leaves during blade development and dehydration.

**Results:**

By combining transverse relaxation maps with a classification scheme, we were able to distinguish specific zones of areoles and veins. The tissue heterogeneity observed in young leaves still occurred in mature and senescent leaves, but with different distributions of T_2_ values in accordance with the basipetal progression of leaf blade development, revealing changes in tissue structure. When subjected to severe water stress, all blade zones showed similar behaviours.

**Conclusion:**

This study demonstrates the great potential of Magnetic Resonance Imaging in assessing information on the structure and water status of leaves. The feasibility of *in planta* leaf measurements was demonstrated, opening up many opportunities for the investigation of leaf structure and hydraulic functioning during development and/or in response to abiotic stresses.

**Supplementary Information:**

The online version contains supplementary material available at 10.1186/s13007-024-01187-2.

## Introduction

Dicotyledon leaf anatomy is complex, characterized by a layered structure that enables efficient photosynthesis. Veins are embedded in parenchyma tissue composed of palisade and spongy parenchyma encased by thin and transparent layers of epidermal cells. The palisade layer, which is rich in chlorophyll, consists of elongated, relatively tightly packed thick-walled cells arranged in several rows, while the spongy layer consists of rounded thin-walled cells surrounded by extensive air spaces, allowing carbon dioxide to diffuse through the leaf and increase its surface area. In the mesophyll outside the xylem, the spongy layer also plays a greater part than the palisade layer in the transport of water in liquid or vapour state to stomata [1]. The venation network in the leaf gradually decreases in size from the petiole to evaporation sites within the leaf [2], ensuring efficient water and nutrient transport to each cell, along with that of photosynthates. Leaf anatomy plays a central role in the leaf hydraulic system [3]. Leaf hydraulic conductance (K_leaf_) is determined by hydraulic conductance levels both inside (K_x_) and outside (K_ox_) the xylem [2, 4], which are related to macroscopic and microscopic structural traits within the venation and areole network [1, 5]. Leaf development or abiotic stress can alter K_leaf_, through its effects on K_x_ and/or K_ox_, affecting for instance the number and dimensions of vein cells [2], the vein hierarchy (i.e. first-, second-, and third-order veins, as well as higher-order veins categorized as minor veins), the ratio of vein length to leaf surface area [4], vein tapering (i.e. narrowing in diameter along the vein length) and leaf surface area [2]. The macroscopic traits of leaf architecture (e.g. vein topology, hierarchy) are usually studied using chemical clearing followed by leaf scanning [5–7], while mesophyll width and cell size are usually estimated from micrographs [1, 7]. While both methods provide reliable information, they remain invasive and result in the loss of leaf material. Microscopy also requires use of multiple replicates to describe heterogeneous tissues due to its limited field of view, providing only localised information. Other techniques have also been proposed to investigate the structural traits of large leaf samples or whole leaves, including X-ray [8, 9], neutron imaging [10], time domain nuclear magnetic resonance (TD-NMR) and magnetic resonance imaging (MRI).

NMR relaxometry offers an efficient and non-invasive way to characterize water dynamics and provide access to cell and tissue structure through subcellular water distribution [11]. In the main cell compartments of plant tissues, water is characterized by different transverse (T_2_) and longitudinal (T_1_) relaxation times governed by water mobility, the chemical exchange of water protons with solutes and solid surfaces, and the diffusion exchange of water molecules between compartments. Diffusion exchange is generally relatively slow, resulting in a multi-exponential relaxation signal that reflects water compartmentalization and is influenced by membrane properties and compartment size [12]. From measurements of NMR relaxation times, it is possible to evaluate water status and distribution at subcellular level, cell/vacuole dimensions and membrane permeability/integrity. A number of studies have used TD-NMR to investigate the structure and hydraulic functioning of plant tissues, only a few of which investigated leaves [13–17]. In studying the leaves of winter oilseed rape (WOSR, *Brassica napus* L.), transverse NMR relaxation parameters proved useful for the monitoring of developmental structural changes [16]. In young leaves, the relaxation signal makes it possible to distinguish the water phase in vacuoles, chloroplasts and cell wall/starch granules. In addition to this, the transverse relaxation signal allows the vacuole water pools in the large and small cells located in palisade and spongy tissues respectively to be differentiated in mature and senescing leaves [17]. Although it makes possible such valuable insights into changes in tissue characteristics, TD-NMR nevertheless requires tissue sampling. Magnetic resonance imaging, on the other hand, offers a non-invasive way to produce transverse relaxation maps at the whole-leaf scale, along with macroscopic structural information. However, the particular structures and shapes of leaves present a considerable challenge in MRI imaging. Because leaves are flat, thin and rich in air, they contain only a small amount of water, resulting in a low signal-to-noise ratio (SNR). In addition, the presence of numerous air–water interfaces leads to susceptibility-induced inhomogeneities that affect the MRI signal. Although decreasing the echo time and magnetic field helps to overcome the latter problem, the low magnetic fields decrease the SNR. Last, commercial radiofrequency (RF) receiver coils are not designed to fit the flat shape of most leaves. Due to these technical difficulties, only a limited number of MRI studies have been performed on whole leaves. These studies [18, 19] were largely confined to achieving a visualization of the leaf architecture using high-resolution morphological images acquired at high fields (7 and 9.4 Tesla). To the best of our knowledge, only Sardans et al. [20] have proposed the use of spatially resolved T_2_ measurements of leaves in order to access information on water status of leaves under water stress, using a 7 T MRI imager. In this study, which monitored long-term *in planta* dehydration in evergreen oak (*Quercus ilex* L.) leaves, T_2_ was consistently higher in the major veins than in the areoles. While T_2_ values for the veins decreased from the leaf base to the distal region according to vein size, the values for the areoles bore no relationship to their position in the leaf blade. Water stress induced a slight increase in T_2_, but only from the fifth week onwards and without inducing any differences between areole zones.

The purpose of the present study was to evaluate the use of MRI as a means to assess, at both tissue and whole-leaf levels, the changes that occur in the structure and water status of WOSR leaves during leaf development and dehydration. To this end, we aimed to demonstrate that mono-exponential transverse relaxation maps obtained by MRI can assess the structural changes in leaf tissues revealed by TD-NMR [17] and to evaluate the possibility of using this method to account for the effect of development and stress at the leaf blade scale. We used a low-field (1.5 Tesla) whole-body imager equipped with a small RF receiver coil that provided a sensible T_2_ mapping compromise between the need for a sufficiently high SNR ratio and the reduction of artefacts caused by the air–water interfaces in the leaves. This whole-body MRI scanner has a large tunnel, making it possible to introduce whole plants in pots and to image leaves without first removing them from the plants. We calculated mono-exponential T_2_ at the voxel level, applying a classification scheme to establish possible heterogeneities in the tissues and leaf zones. The effects of leaf development on water status and distribution were investigated in leaves at three developmental stages and dehydration kinetics were studied for one stage. In both cases, leaves were detached from the plant in order to simplify the MRI experimental protocol. Last, the MRI experiment was performed on leaves attached to the plants (*in planta*) to demonstrate the feasibility of the method.

## Methods

### Experimental design

Three experiments were performed in order to address the objectives described above: (i) The effects of leaf development on water status and distribution were investigated in young, mature and senescing detached leaves kept hydrated; (ii) Dehydration kinetics were studied in detached mature leaves and (iii) *In planta* experiment was performed on mature leaves attached to the plants.

### Plant materials, growth conditions and experimental design

A total of 50 winter oilseed rape (WOSR, *Brassica napus* L., cultivar Aviso) seeds were individually weighed, and the 25 most uniform seeds were selected and placed in a petri dish on filter paper soaked with water. After 3 days of germination, 20 seedlings were selected based on visual criteria (root development, cotyledon emergence, overall uniformity) and then individually transplanted into mini pots (7 cm × 7 cm surface area × 14 cm height, 0.4 L capacity). The pots were filled with a mixture (ref. 992016F1, Falienor®) of sandy loam soil (40% v/v) and peat (60% v/v) with the addition of clay (40 kg m^−3^) and of NPK (0.7 kg m^−3^ PG-MIX 14–16-18) mixed with a soil solution at a pH of 5.8 ± 0.2. All plants were grown in a semi-controlled greenhouse environment equipped with an air-cooling system, targeting a temperature/humidity/photoperiod of 25 °C / 75% / 14 h of daylight and 18 °C / 90% / 10 h of darkness. Natural light was supplemented as needed to ensure a minimum of 200 µmol m^−2^ s^−1^ of photosynthetically active radiation (PAR) at canopy height. All plants (non-vernalized) were watered manually with fertilized water (0.3% Liquoplant Bleu, 2.5% N, 5% P, 2.5% K) every morning to replenish evaporative loss. Among the 20 plants grown, 6 plants were selected using visual criteria (leaf homogeneity in terms of number, surface and colour) for use in the MRI experiments.

### Leaf stage selection and sampling

**Fig. 1:**
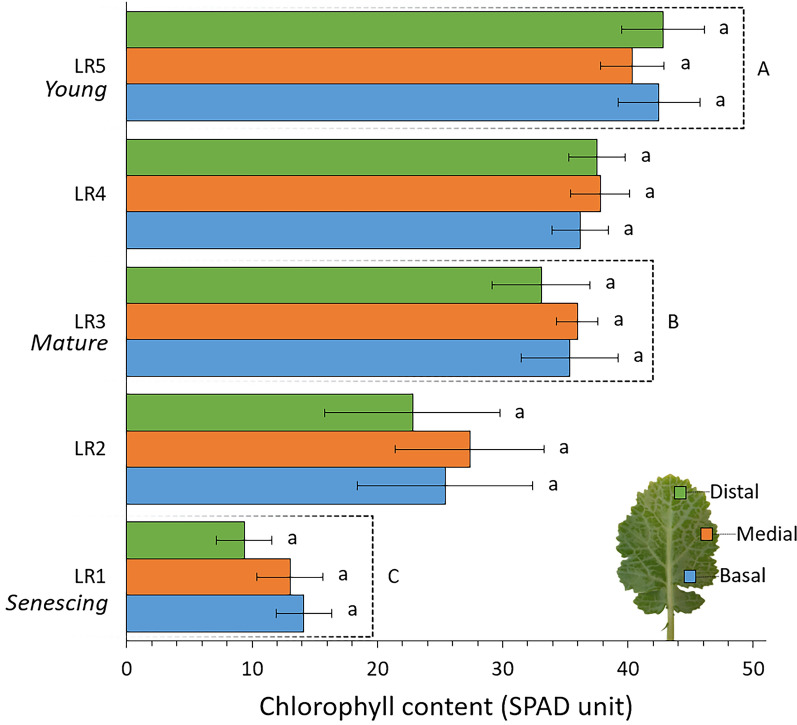
Chlorophyll content in the distal, median and basal leaf regions for young (leaf rank 5), mature (leaf rank 3) and senescing leaves (leaf rank 1). Values are the means ± SD of 3 independent biological replicates. Lowercase letters indicate significant differences in values between leaf regions and uppercase letters indicate significant differences in values between leaf ranks (taking all three regions into account), as determined using ANOVA followed by a Tukey HSD test (p-value < 0.05)

Leaves were selected from plants and sampled at BBCH scale 15 (plants with 5 true leaves in addition to cotyledons). A leaf rank (LR), corresponding to the order of leaf emergence on the plant, was assigned to each sampled leaf from oldest (LR1) to youngest (LR5). The chlorophyll content measurements were performed on all leaves using a SPAD-502 chlorophyll meter (Minolta). All leaves exhibited similar chlorophyll content in their distal, medial, and basal regions (Fig. 1), but the total chlorophyll content decreased significantly as the leaves developed. Young (LR5), mature (LR3) and senescing leaves (LR1) showed significantly different chlorophyll contents and were therefore considered as contrasted leaves for the experiment on leaf development. For each of the three replicates, young, mature and old leaves were taken from the same plant. For the dehydration and *in planta* experiments, only mature leaves were used. Leaves were collected from the plant by cutting across the petiole on the first third of its length, except for the *in planta* experiment, where leaves were not excised. In detached leaves, hydration was maintained during imaging experiments by plunging the basal section of the petiole into a 2 mL microtube of water.

### MRI acquisition and image processing

MRI images were acquired using a whole-body 1.5 Tesla MRI scanner (Magnetom Avanto, Siemens) equipped with a high-resolution wrist array receiver RF coil. Leaves were placed abaxial side up in a specially designed device (Additional file 1: Fig. S1) consisting of two grids separated by a 2 mm gap to allow air circulation. Leaves were held stable in the device by using a weight to exert slight pressure on the upper plate, maintaining a constant gap between the plates. The temperature of the MRI room was kept at 20 °C.

For *in planta* acquisitions, the plant pot was wrapped in plastic film to prevent soil dehydration. The pot was positioned on a base that was tilted to align the leaves with the receiver RF coil while allowing water from the substrate to drain away from the pot. The leaf under investigation was placed in the device described in the previous paragraph (Additional file 1: Fig. S1), while the other leaves of the plant were held away from the device.

The MRI images used to calculate T_2_ maps were acquired using a multi-echo spin sequence (MSE) with 32 echoes, echo time (TE) 5.2 ms, repetition time (TR) 600 ms, bandwidth 450 Hz/pixel, field of view (FOV) 128 mm × 128 mm, and matrix size 160 × 160 and one coronal slice of 10 mm thickness containing the whole leaf thickness. The image spatial resolution was 0.8 mm × 0.8 mm × 10 mm. Four experimental protocols were performed:

1. To study leaf development, MRI MSE images were acquired on young, mature and senescing leaves that were kept hydrated. 12 scans were used to improve the SNR, resulting in acquisition time of 19 min 16 s for each image series. The number of scans were kept the same for the second and third experiments.
2. To exclude any source of signal variation related to leaf evolution or imager instability and to ensure that any differences observed during dehydration were due to this process, MSE images of mature hydrated leaves were acquired over a total of 6 h and 30 min, with a 10-min pause between each acquisition, resulting in an acquisition every 30 min.
3. To study the leaf dehydration kinetics, a baseline image was first acquired for mature leaves supplied with water. The water-filled tube was then removed and images were acquired for a total of 6 h and 30 min, with a 10-min pause between each acquisition, resulting in an acquisition every 30 min.
4. To demonstrate the feasibility of *in planta* imaging and to evaluate the possibility of reducing the acquisition time, MSE images of non-excised mature leaves were acquired using 3 scans, resulting in a total acquisition time of 4 min 48 s.

Three biological replicates were acquired for each experiment.

Mono-exponential T_2_ maps were computed using MATLAB software [21] from the images acquired using the MSE sequence, following the method described in [22]. The latter maximizes the likelihood of the data while imposing spatial regularity on the solutions in order to reduce the effects of noise. A pair of parameters adjusts the weight of the regularization as described in Appendix. They were chosen in order to achieve a compromise between a regularized solution and the preservation of a sufficiently high spatial variations to distinguish, in particular, between the veins and the areoles. To reduce the complexity of the information and facilitate image interpretation, T_2_ maps were post-processed by the K-means algorithm [23] using ImageJ software [24] which, when assigned a number of classes, clusters voxels with similar parameters. Our choice of five of classes aimed to both simplify the visualization and retain sufficient detail for the structures to be observable. This number of classes was used for all the experiments. Clustering was conducted independently for each leaf, prioritizing significant differences in T_2_ between various regions of the leaf blade rather than absolute T_2_ values. Once classification had been carried out, each class was assigned a number and a colour in ascending order of T_2_ values (1-purple, 2-cyan, 3 dark orange, 4-light orange, 5-yellow). In this post-processing step, leaflets were excluded from the analysis because they are individual structures whose evolution is not expected to be coordinated with that of the rest of the limb.

### Data analysis

Chlorophyll contents were expressed as the mean ± SD of 3 independent biological replicates. Statistical analysis of chlorophyll content was conducted using a one-way analysis of variance (ANOVA) followed by a post-hoc Tukey's HSD test for multiple pairwise comparisons to determine significant differences (p-value < 0.05) between regions (distal, medial and basal) and leaf ranks. Statistical analyses of MRI results were conducted based on the T_2_ values of each class to examine the effect of developmental stage within the same plant and the effects of leaf dehydration for individual leaves. As the T_2_ values for each class did not follow a normal distribution, we analysed their median values. A one-way analysis of variance (ANOVA) was used to determine significant differences (p-value < 0.05) between classes of the same leaf and between leaf ranks and dehydration times for each class. Multiple pairwise comparisons were conducted using a post-hoc Tukey's HSD test. The statistical analyses were performed using RStudio software [25].

## Results

### Leaf development

**Fig. 2:**
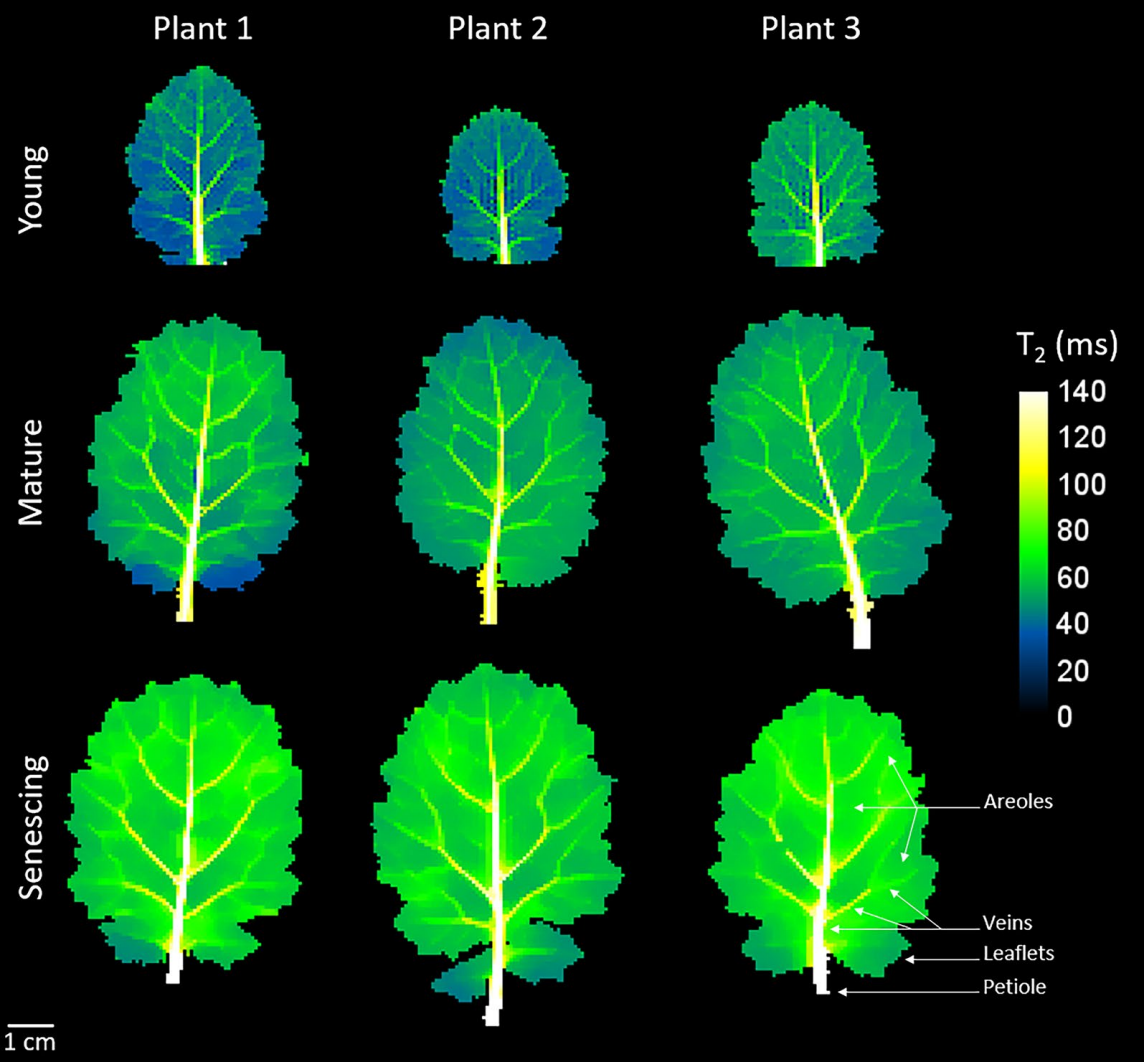
Transverse relaxation (T_2_) maps of 3 young (leaf rank 5), 3 mature (leaf rank 3) and 3 senescing (leaf rank 1) leaves. A water reservoir, connected to the basal section of the petiole to ensure leaf hydration, is not shown on the image

Mono-exponential T_2_ maps of young, mature and senescing leaves from 3 plants are shown in Fig. 2. Leaf area was significantly higher in mature leaves (26.2 ± 3.1 cm^2^) than in young leaves (10.6 ± 1.6 cm^2^) and was similar in senescing (29.3 ± 3.0 cm^2^) and mature leaves (Additional file 2: Fig. S2). As a first observation of Fig. 2, in young leaves, areoles were characterized by T_2_ values of about 40 ms. The main veins running from the petiole to the apex were easily distinguished from the areoles by their longer T_2_ values, which decreased from the basal zone (~ 140 ms) to the distal zone (~ 80 ms). Since their T_2_ values were higher than those of the areoles, second and third order veins could also be identified. The same leaf morphological features were observed in the T_2_ maps of mature and senescing leaves, with T_2_ values for the areoles and main veins increasing with leaf development. In fact, T_2_ values in the areoles were ~ 50 ms and ~ 60 ms for mature and senescing leaves respectively.

**Fig. 3:**
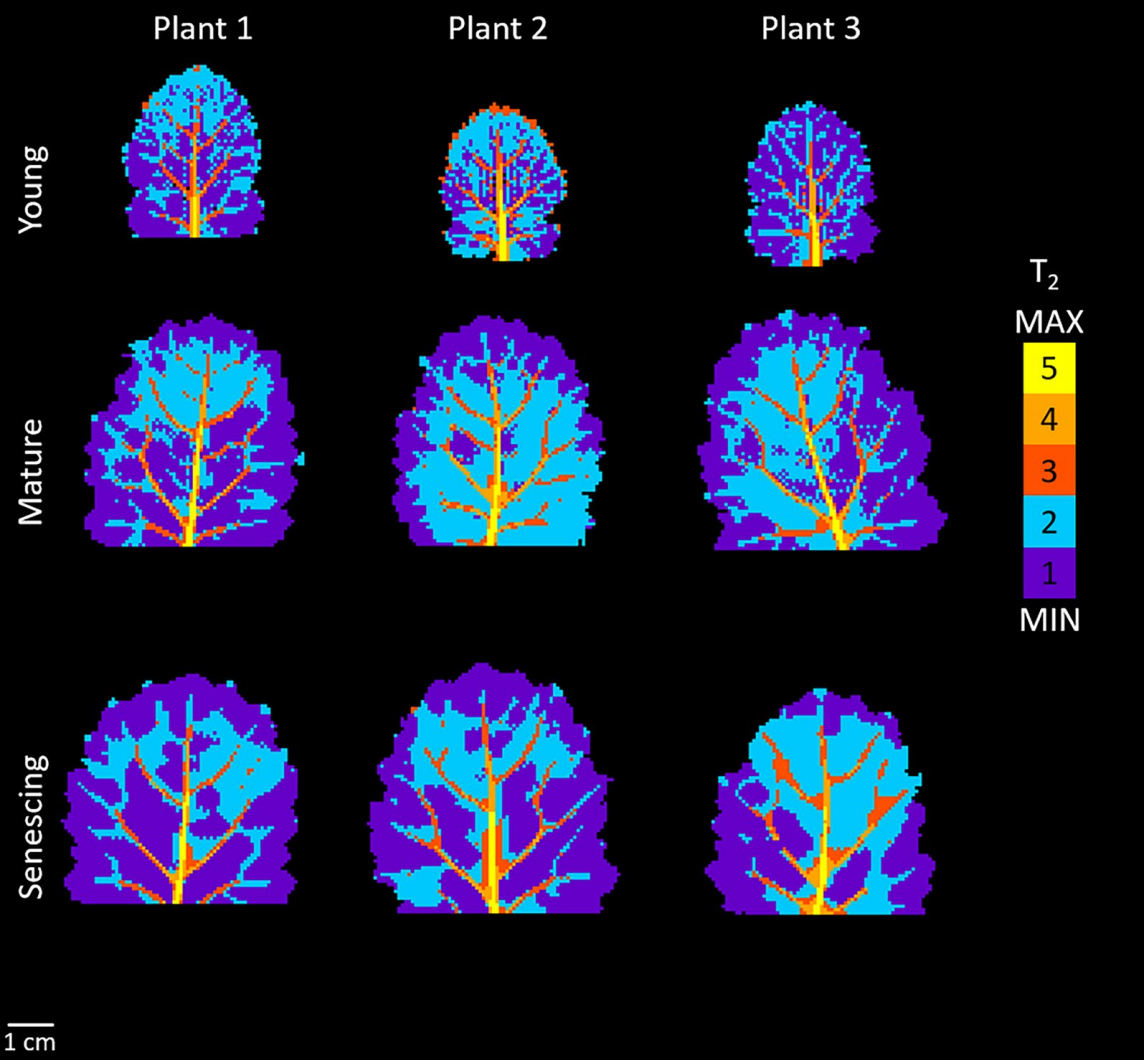
Voxel-based classifications applied to the transverse relaxation (T_2_) maps of the 3 young (leaf rank 5), 3 mature (leaf rank 3) and 3 senescing (leaf rank 1) leaves shown in Fig. 2

In order to establish possible heterogeneities in the tissues and leaf zones and to statistically analyse the effects of leaf development on T_2_ values, we applied a classification scheme on T_2_ maps. Figure 3 shows the results when the T_2_ maps from in Fig. 2 are clustered, where the scale represents the rank of each class according to its T_2_ value. From lowest to highest T_2_ values, the first two classes were located in the areoles (purple, cyan) and the other three classes corresponded to veins (dark orange, light orange and yellow). In young leaves, it was not possible to establish a general pattern of spatial distribution for purple and cyan voxels (classes 1 and 2 respectively), although the two classes appear to be spatially coherent, corresponding to specific leaf zones in the areoles. Voxels in the purple class, characterized by the lowest T_2_ values, tended to be located at the edges of mature leaves. This finding was confirmed in senescing leaves, where the lowest T_2_ values also extended to most of the basal zone of leaf areoles. It should be noted that the mature leaf of Plant 1 displayed a pattern similar to those of the senescing leaves. The distal portion in dark orange (class 3), the central portion in light orange (class 4) and the basal portion of the primary vein in yellow (class 5) could be clearly observed in all leaves, regardless of developmental stage. The secondary veins fell into the light and dark orange clusters, while voxels corresponding to tertiary veins appeared in dark orange or cyan. The classified T_2_ maps appeared quite symmetrical in relation to the primary vein.

**Fig. 4:**
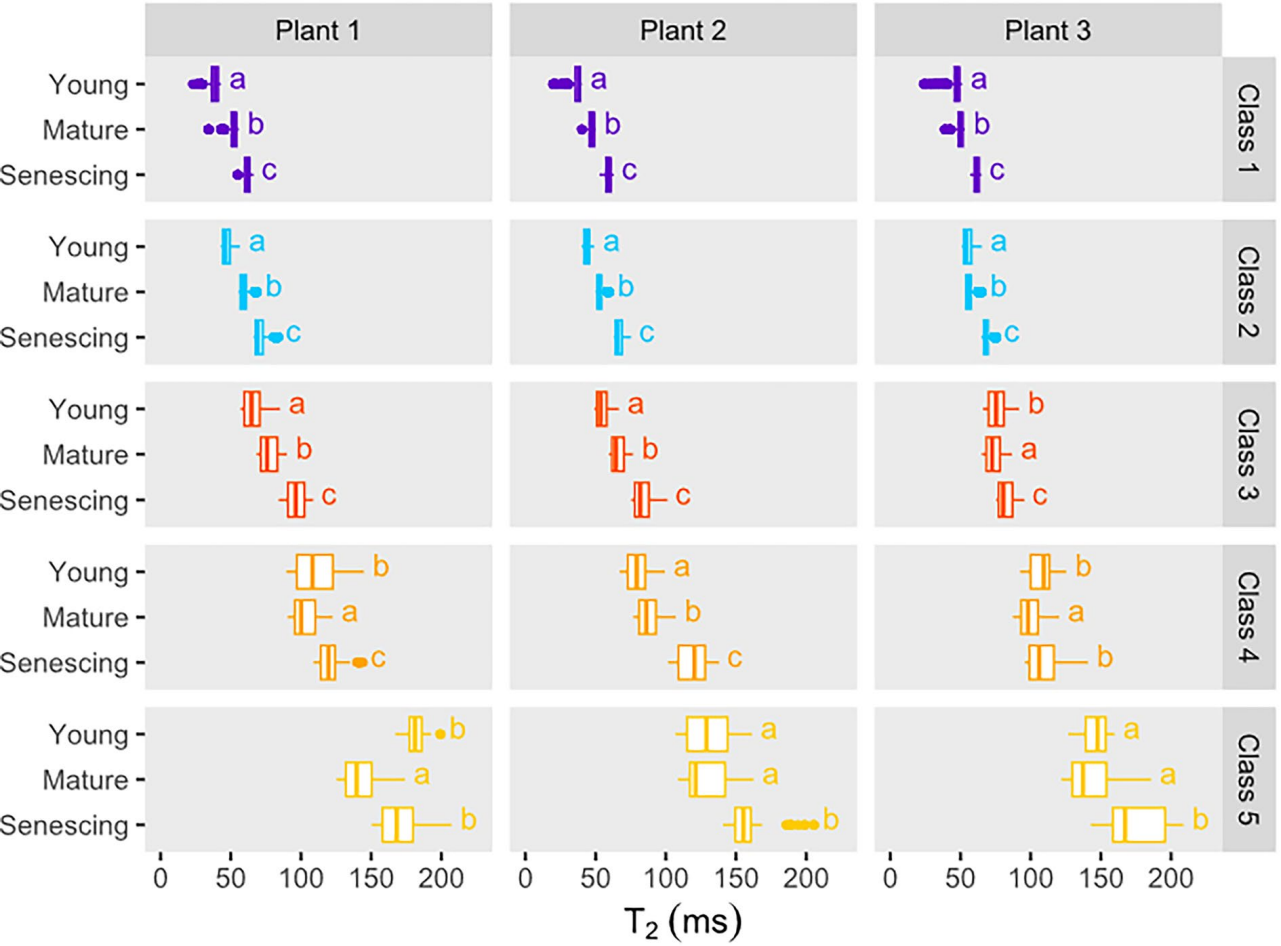
Box plot of transverse relaxation (T_2_) values in the five classes for the young (leaf rank 5), mature (leaf rank 3) and senescing leaves (leaf rank 1) of each plant. Letters indicate significant differences between leaf ranks for each class and plant, as determined using ANOVA followed by a Tukey HSD test (p-value < 0.05)

The evolution of median T_2_ values by developmental stage for each class of the 3 plants is shown in Fig. 4. In order to prevent effects of possible slight variations in the developmental stage of leaves of the same rank but from different plants [17], the numerical values are represented for each biological replicate.The T_2_ values for the two first classes (purple and cyan) located in the areoles increased with age, as did the T_2_ values for the dark orange class 3, which corresponded to the smaller veins (the distal part of the main vein and the secondary and tertiary veins). In the case of yellow class 5, corresponding to the basal part of the main vein, T_2_ values increased significantly from mature to senescing leaves. The same trend was observed for the light orange class 4, which mainly corresponded to the middle section of the main vein and the secondary veins.

### Dehydration

**Fig. 5:**
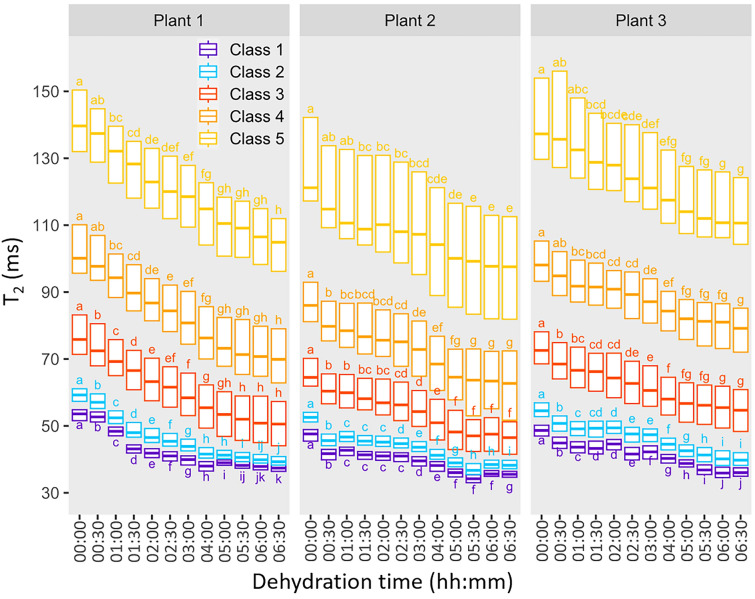
Box plot of transverse relaxation (T_2_) values of the five classes for the mature (leaf rank 3) leaf of each plant. Note that minimum, maximum, and outlier values are omitted from each box plot to avoid overlap. Letters indicate significant differences between dehydration times for each class and plant, as determined using ANOVA followed by a Tukey HSD test (p-value < 0.05)

When the mature leaves were kept hydrated by a water reservoir connected to the petiole, the T_2_ maps remained stable over the 6 h 30 min period corresponding to the dehydration process (Additional file 3: Fig. S3, p-value < 0.05), demonstrating that leaf evolution and imager instability could be excluded as possible sources of signal variation. When leaf dehydration was initiated by removing the water reservoir from the petiole, this resulted in a decrease in T_2_ in both areoles and veins. To quantify the heterogeneity of the areolar tissues and veins and the evolution of these heterogeneity as the dehydration period progressed, the five classes specified for the T_2_ maps at the zero time point (Fig. 3, middle line, mature leaf) were applied to all subsequent images acquired during dehydration. The median T_2_ values for each of the five classes showed a progressive decrease (Fig. 5) up to the 5-h time point, after which T_2_ remained stable in almost all classes. For the same reasons as in Fig. 4, T_2_ values are represented for each biological replicate. The decrease in T_2_ was similar for all five classes. Significantly different values were present between classes (*p-value* < 0.05) at all measuring points, demonstrating that the zones defined at the baseline could be distinguished throughout the dehydration period.

### *In planta* leaf imaging

**Fig. 6:**
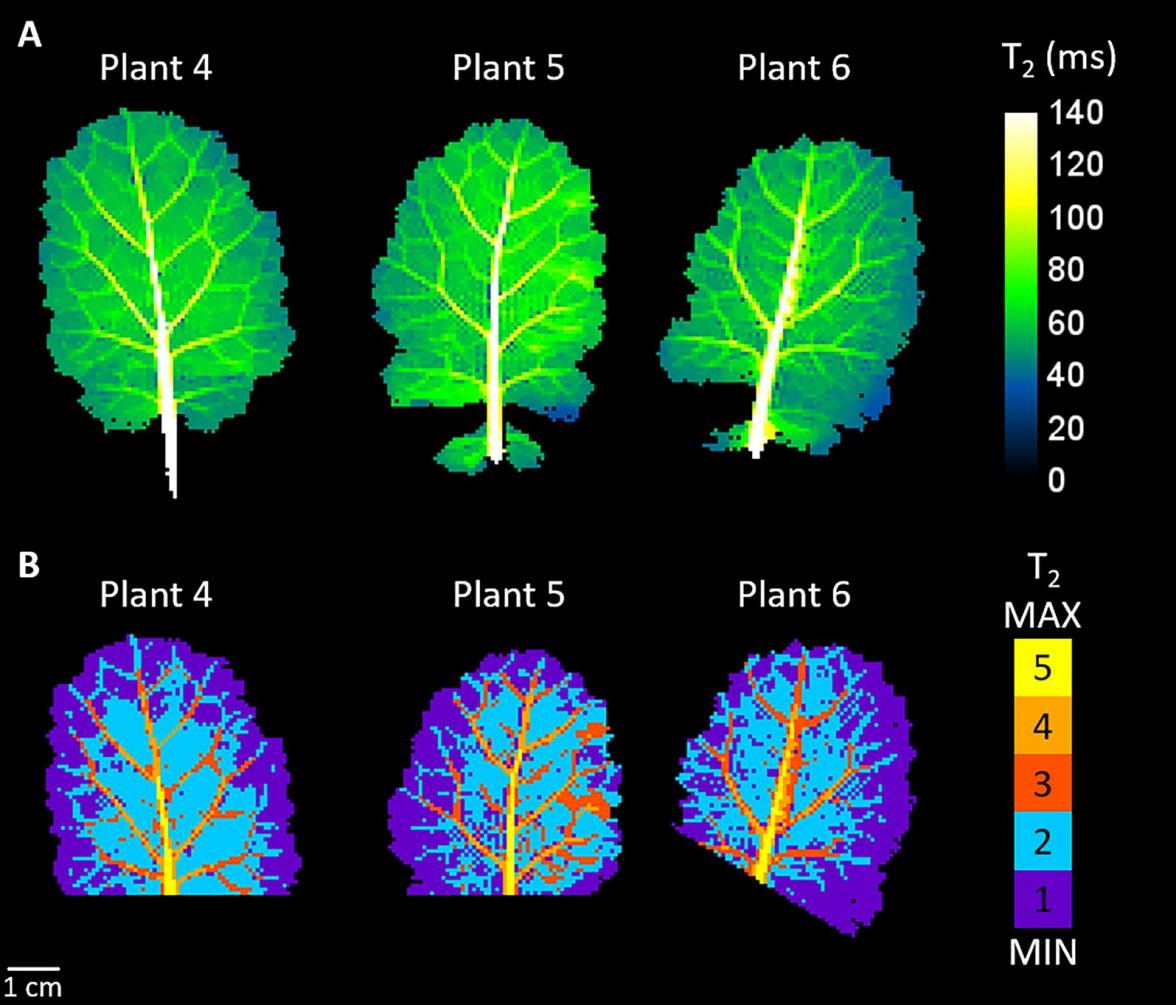
Transverse relaxation (T_2_) maps of 3 mature (leaf rank 3) leaves computed from *in planta*-acquired MRI images (**A**) and their corresponding classifications (**B**)

T_2_ maps of mature leaves (A) computed from *in planta*-acquired MRI images and the corresponding clustering results (B) are shown in Fig. 6. As in excised leaves, the purple and cyan classes (1 and 2 respectively), with the lowest T_2_ values, were located in the areoles, while the other three classes were located in the veins (dark orange, light orange and yellow). Pixels in the purple class (class 1) were located at the edge of the leaves, while the cyan class (class 2) occupied the central parts of the areoles. The veins mainly consisted of three spatially coherent classes (yellow, light orange, and dark orange). From the first to the fifth class, the median T_2_ values for each class were 51 ms, 60 ms, 78 ms, 109 ms and 165 ms. These values were roughly the same as those found in excised mature leaves. Additionally, these results, obtained with a shorter acquisition time (4 min 48 s), demonstrate the possibility of being able to measure T_2_ maps *in planta* at rates of the order of just a few minutes.

## Discussion

This MRI study, based on T_2_ mapping at low magnetic field, made possible investigation at blade level of the changes in the structure and water status of oilseed rape leaves that occur during leaf development and dehydration. Its findings and the relevance of these MRI approaches are discussed in the following sections.

According to Sack and Scoffoni [2], leaf growth consists in a phase of cell proliferation and low expansion, followed by a phase of strong increase in leaf blade area, mainly due to cell elongation, although cell division continues. This process occurs basipetally, in that cell elongation in the mesophyll occurs first in the distal region and then extends towards the basal region. It may lead to variations in tissue development, this being more advanced in the distal region than in the proximal and basal regions. The vascular tissues form at different times; while major (first to third order) veins develop in the earlier stages of leaf ontogenesis, more minor veins (higher than third-order) are formed later, during a rapid expansion phase [26], and thus quickly reach their maximum size. In this study, MRI was performed at three contrasted stages of leaf development—young (LR5), mature (LR3) and senescing (LR1). The differences between the LR5 and LR3 leaves corresponded to the transformation from young to mature leaves, comprising a rapid three-fold expansion in leaf area until the mature leaf attains its final size and switches status from sink to source. By contrast, the transition from mature to senescing leaves was not associated with an increase in leaf area but with a decrease in leaf chlorophyll content. Regardless of leaf development, the classification scheme applied to the T_2_ leaf maps made it possible to identify five spatially coherent classes of voxels reflecting differences in water status both within and between the areoles and veins. The leaf blades were characterized by two classes to describe areoles (classes 1 and 2) and three other classes which mainly corresponded to vascular tissue. It should be noted that although classes 1 and 2 were predominantly assigned to areoles, class 2 also included the minor veins (orders higher than 3) that were not detectable in classes 3 to 5 (Fig. 3). In mature and senescing leaves, the edges and the basal zone of the leaf generally differed from the other parts of the areole, probably due to the particular structure of these zones, with cells at the edge of the leaf not fully expanded due to the thinness of the leaf tissue.

The increase in areole T_2_ values from young to mature leaves can be explained by the increase in cell and vacuole sizes that occurs as cells expand during this period, since T_2_ is sensitive to compartment size [27]. The spatial differentiation of class 2 in the distal region (except at the edges of the leaf, see previous paragraph) can be explained by the early cessation of cell proliferation in the distal region of a growing leaf, while cells in the proximal region continue to divide for longer [28]. This means that areoles in the distal region show signs of earlier maturation than areoles in the basal region [28], as reflected here by the higher T_2_ values for class 2 compared to class 1. The increase in T_2_ observed here between mature and senescing leaves is documented in the literature for NMR studies of oilseed rape leaves [16, 17], tobacco [29] and potato [30]. In oilseed rape [17], changes in the component of the multi-exponential transverse relaxation signal associated with the vacuole and its division into two fractions occur at sink-to-source transition. Thus, two vacuole components are to be observed in mature and senescing leaves, correlated with specific changes occurring in the palisade and spongy parenchyma. These physiological changes involve significant enlargement of palisade cell vacuoles during senescence, in contrast to the spongy parenchyma cells which evolve very little. Cell enlargement was shown to occur in the direction of the leaf thickness rather than laterally. This observed increase in cell size from mature to senescent leaves can be explained by a loosening of the cell wall [31, 32] associated with water influx induced by senescence. Our results, which showed an increase in mono-exponential T_2_ values for the areoles (classes 1 and 2) between mature and senescing leaves (Fig. 4), while leaf surface values remained stable (Additional file 2: Fig. S2), were consistent with these NMR studies [16, 17]. The higher T_2_ values for the major veins (primary, secondary and tertiary veins, classes 3 to 5) compared with those for the areoles and minor veins are consistent with the fact that the xylem and phloem cells of major veins have larger diameters than minor veins and the mesophyll cells of areoles, but lower density [3]. Tapering of the major veins [2] was reflected in the decrease of T_2_ values along their length (Fig. 3). Thus, the primary vein in the distal region has a T_2_ similar to the secondary veins identified in class 4, and the same is true of the relationship between secondary and tertiary veins in class 3. The classification described for primary veins therefore appears to reflect their size. We observed an increase in T_2_ values for secondary and tertiary veins (classes 3 and 4) from young to mature leaves (Fig. 4), which is in line with their continuous diameter increase during leaf expansion [26].

Since water enters the leaf via the petiole [2], the well-hydrated conditions in this study were produced by inserting the petiole in a water reservoir, while the removal of the reservoir cut off the water supply, thereby simulating severe leaf tissue dehydration. This led to a progressive decrease in T_2_ for all the five classes defined on the well-hydrated leaves, consistent with the results obtained for bean and poplar leaves subjected to dehydration a few hours after being cut from the plant or to osmotic stress [15], and with those from the NMR relaxometry study of oilseed rape leaves subjected to long-term water stress (Boulc’h *et al.* [33], *submitted to Plant Cell & Environment*). The water loss that causes the decrease in T_2_ can result from both cell shrinkage and an increase in the solute concentrations of the aqueous solutions contained in cell compartments. It may also be due to the increase in membrane permeability, regulated by the activity of aquaporins, that facilitates the diffusional exchange of water molecules between cell compartments and maintains cellular expansion during stress. Conversely, it is possible that an increase in T_2_ values may be observed when, as van der Weerd *et al.* [27] suggests, drought causes membranes to become less permeable to water exchange in order to retain the cellular water within cell compartments. This last hypothesis was proposed by Capitani *et al*. [15] to explain an increase in T_2_ after 24 h of leaf dehydration. Similarly, Sardans *et al.* [20], has attributed the increase in leaf areole T_2_ values once watering was interrupted for holm oak to a decrease in tonoplast membrane permeability in response to water stress. In the latter study, taking into consideration the time interval between measurements (~ 40 days), it is possible that the increase in T_2_ was at least partly due to changes in structure associated with leaf development rather than dehydration. Figure 5 shows that leaf dehydration was not impacted by differences between the tissues, as the T_2_ values for the five classes decreased in a similar way. We found in a previous study (Boulc’h *et al.* [33], *submitted to Plant Cell & Environment*) that the adaptive response to water stress in oilseed rape was strongly modulated by leaf developmental stage. In plants subjected to severe soil drought, young leaves showed no signs of cell dehydration and compensated for water deficit through structural changes and osmotic adjustment. By contrast, mature leaves were unable to counteract water deprivation leading to cell dehydration. On the basis of the leaf blade heterogeneity caused by the basipetal progression of mesophyll development described above, it could be expected that dehydration would affect the distal zone earlier, but this was not the case. Interestingly, Sardans et al. [20] have shown that the distal part of the leaf loses water faster when water deficit reaches a certain, relatively high, threshold.

Few NMR [15–17, 29, 30, 34] or MRI [20] relaxometry studies have been performed on leaves or leaf samples from different species. Direct comparison of the relaxation parameters measured in these studies is complex, since they depend not only on the structure and composition of a leaf tissue, but also on the experimental conditions (magnetic field strength and experimental protocol). Nevertheless, to provide a broader context for our results, it is of interest to discuss the nature and relevance of the information that can be accessed using these different approaches. The main advantage of MRI over NMR is that it allows non-invasive transverse relaxation mapping of intact whole leaves, making it possible to detect variations in tissues and to study a leaf’s kinetics. However, since the T_2_ for leaf tissues is relatively short, it is difficult to set the TE to a sufficiently short value for the relaxation curve to be sampled with enough precision to fit a multi-exponential relaxation model, known to be the optimal model for describing transverse relaxation in plant tissue, including leaves. Consequently, a mono-exponential T_2_ is calculated instead, providing information that averages the contributions from the different pools of water contained in the leaf thickness. A further possible difficulty arising from the MRI approach is that the diffusion of water molecules through local magnetic field gradients induced by the relatively high air content of the leaf areoles tends to reduce not only the absolute T_2_ values, but also their variation ranges, thus potentially masking or biasing differences between the tissues. In the present study, by performing MRI experiments at a low magnetic field (1.5 Tesla) and using a TE of 5.2 ms, we observed relatively high T_2_ values (several tens of milliseconds, Figs. 2, 3, 4) compared to those reported by Sardans et al. [20] at 7 Tesla (~ 15 ms in areoles and 20–30 ms in veins). We were also able to reveal tissue heterogeneities in well-hydrated leaves (Figs. 3 and 4), unlike Sardans et al. [20].

For the estimation of the T_2_ maps, spatial regularization was used to overcome noise. Given the relatively low SNR in this study, this regularization was necessary to obtain voxels that, after clustering, fell into spatially coherent classes with similar T_2_ values, making it easier to analyse their spatial heterogeneity. The degree of regularization involved is governed by the choice of two parameters [22]. A first approach is to consider that one parameter regulates the overall weight of the regularization, while the other controls the preservation of important transitions between two voxels, such as a transition between one vein and one areole. This pair of parameters could induce either under- or over-regularization if not chosen properly. For this particular application, one possible bias lay in the fact that high T_2_ values in the veins would propagate into the limb zone. The regularization parameters were set so that this bias was minimal. For the leaf development experiment, the average absolute differences, expressed in relative values, between the mean T_2_ values for classes 1 and 2 and estimated with and without spatial regularization were, respectively, 3.8 ± 1.6% and 2.2 ± 1.7%. These differences could be considered to be negligible, demonstrating that the regularization parameters had been properly tuned. Interestingly, the fact that the results can be spatially regularized in this way opens up the possibility that acquisition times might be shortened by reducing the number of scans without loss of quality in computation, as was observed for the *in planta* experiments (3 scans).

The spatial resolution of the MRI images was 0.8 × 0.8 × 10 mm^3^. This meant that, depending on structure sizes, one voxel was likely to contain several tissues. For leaf veins with a diameter of less than 0.8 mm, a voxel would contain both the vein and mesophyll tissue, suggesting that its signal, and therefore the measured T_2_, would represent a weighted average of the T_2_ of the two tissues. It is possible to increase the spatial resolution (decreasing the size of the voxel) but at the cost of increasing acquisition time or reducing the SNR. It is interesting to note that, in the case of leaves, which are very thin, this partial volume phenomenon does not occur in the direction of the slice thickness.

In line with a handful of previous works such as Sardans et al. [20], this study confirms MRI’s strong potential for the *in planta* non-invasive and non-destructive continuous characterization of leaf water status and functioning. A number of improvements could optimise plant conditions during short- and long-term studies. Among them, the acquisition time for T_2_ mapping could be kept below 5 min, as shown in the *in planta* study (Fig. 6), or even reduced for the study of rapid changes in leaves. Also, the illumination of the plant can be easily performed by introducing a light source that replicates natural light [35], thereby ensuring optimal leaf functioning. It would be interesting to quantify the morphological descriptors of the vascular network during leaf development. To achieve this successfully, given that vascular bundles in dicotyledonous leaves gradually decrease in size, spatial resolution should be increased to allow accurate detection of higher order veins.

## Conclusions

This study demonstrates the great potential of MRI in assessing information on the structure and water status of leaves. Mono-exponential T_2_ maps were shown relevant to assess the structural changes previously revealed by multi-exponential TD-NMR relaxation signal. The combination of T_2_ relaxometry with the classification scheme succeeded to reveal tissue heterogeneity in leaf blades. The tissue heterogeneity was observed in young, mature and senescent leaves, but with different distributions of T_2_ values in accordance with the basipetal progression of leaf blade development, revealing changes in tissue structure. When subjected to water stress, all blade zones showed similar behaviours. The feasibility of *in planta* leaf measurements was demonstrated, paving the way for non-invasive and non-destructive measurements of physiological changes in leaves during development and/or in response to abiotic stresses.

### Supplementary Information

**Additional fle 1:** **Figure S1.** Device for leaf MRI imaging. A, 3D view schema and B, picture of a leaf placed in the device inside the wrist coil. C, a microtube containing water was connected to the leaf petiole when maintaining hydration of excised leaves was required. In B and C, weight exerting slight pressure on the upper plate used to hold leaves stable in the device is shown.

**Additional fle 2:** **Figure S2.** Leaf area for young (leaf rank 5), mature (leaf rank 3) and senescing (leaf rank 1) leaves. Values are the means±SD of 3 independent biological replicates. Letters indicate signifcant variations in values between leaf ranks, as determined using ANOVA followed by a Tukey HSD test (p-value<0.05).

**Additional fle 3:** **Figure S3.** Transverse relaxation (T2) maps of a mature leaf (leaf rank 3) over a time period of 6h30min.

## Data Availability

All materials and data analysed in this study are available from the corresponding author.

## Appendix

The signal in voxel $x$ at echo time $t$ was modelled as an exponential decay governed by amplitude $I(x)$ and relaxation time ${T}_{2}\left(x\right)$, which writes:

$$s\left(x,t\right)=I(x){e}^{-t/{T}_{2}(x)}$$

The measured signal, $y\left(x,t\right),$ is affected by noise, considered as following a Rician distribution, ${P}_{{\text{Ric}}},$ typically encountered in MRI images which are actually module images computed from one real and one imaginary part, each being corrupted by a non-correlated Gaussian centered noise of variance ${\sigma }^{2}$. $\sigma$ can be measured in the images from the voxels in the background where no signal is present as explained in [22].

In order to estimate in each voxel $I\left(x\right)$ and ${T}_{2}\left(x\right)$, the following criteria $J$ was minimized regarding these unknowns:

$$J=\prod_{x=1}^{X}\prod_{t=1}^{T}{P}_{{\text{Ric}}}\left(y(x,t) | s(x,t),{\sigma }^{2}\right)+\beta \sum_{x=1}^{X}\sum_{v\in V(x)}\left(\psi \left(I\left(x\right)-I(v)\right)+\psi \left({T}_{2}\left(x\right)-{T}_{2}(v)\right)\right)$$

with

$$\psi \left(u,v\right)=\frac{{\left(u-v\right)}^{2}}{u+v+\delta \left|u-v\right|}$$

This criteria takes into account all together the $X$ voxels and $T$ echo times. The first term stands for the likelihood of the signal $y(x,t)$ regarding the model $s\left(x,t\right)$ and the Rician noise, the second term imposes spatial regularity in the solutions, $I(x)$ and ${T}_{2}\left(x\right),$ in order to reduce the effects of the noise. Thus, the criteria represent a balance between the adequacy of the data to the model and a regular spatial solution. $V(x)$ corresponds to the set of the voxels that are in the neighborhood of $x$. For this work we used the 8 nearest voxels. The function $\psi$ was chosen so as to provide smoothing while enabling the preservation of large transitions between different zones, such as areolas and veins. Two parameters $\beta$ and $\delta$ governs the importance of spatial regularization. Their optimal values depend on the object observed as well as the signal to noise ratio, so they have to be tuned regarding the application. They were chosen among a large set of couples of values by visual assessment of the intensity of regularization and preservation of structures in the estimated ${T}_{2}$ maps. The actual values were 57 for $\beta$ and 250 for $\delta$. The minimization of $J$ was done using the method described in [22] implemented in Matlab.

## Abbreviations

K_leaf_: Leaf hydraulic conductance
K_x_: Leaf hydraulic conductance inside the xylem
K_ox_: Leaf hydraulic conductance outside the xylem
NMR: Nuclear magnetic resonance
MRI: Magnetic resonance imaging
T_2_: Transverse relaxation time
T_1_: Longitudinal relaxation time
TD-NMR: Time-Domain NMR
WOSR: Winter oilseed rape
SNR: Signal-to-noise ratio
RF: Radio frequency
PAR: Photosynthetically active radiation
LR: Leaf rank
MSE: Multi-echo spin sequence
TE: Echo time
TR: Repetition time
FOV: Field of view
