# Supplementary figures and images for: Quantitative MRI imaging of parenchyma and venation networks in Brassica napus leaves: effects of development and dehydration
Source: Plant Methods. 2024 May 13;20:69. doi: 10.1186/s13007-024-01187-2 (PMC11089671; doi:10.1186/s13007-024-01187-2)

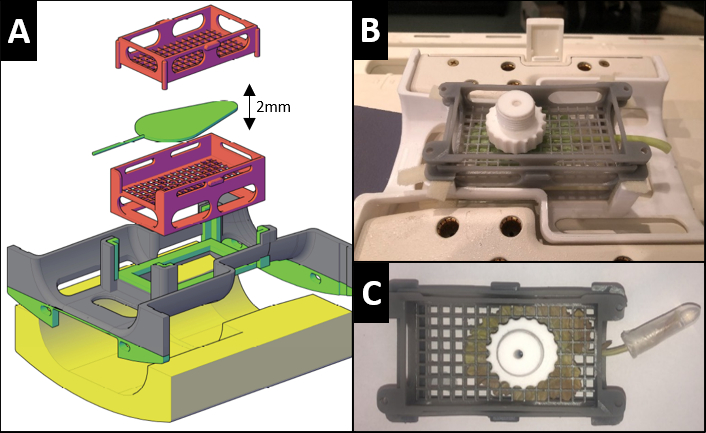

Supplement: Supplementary file 1 — Additional fle 1: Figure S1. Device for leaf MRI imaging. A, 3D view schema and B, picture of a leaf placed in the device inside the wrist coil. C, a microtube containing water was connected to the leaf petiole when maintaining hydration of excised leaves was required. In B and C, weight exerting slight pressure on the upper plate used to hold leaves stable in the device is shown. [file 13007_2024_1187_MOESM1_ESM.jpg]

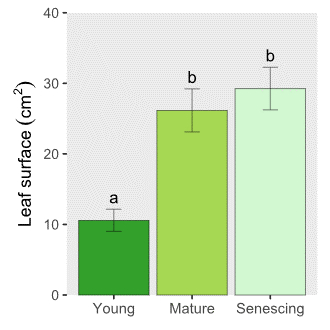

Supplement: Supplementary file 2 — Additional fle 2: Figure S2. Leaf area for young (leaf rank 5), mature (leaf rank 3) and senescing (leaf rank 1) leaves. Values are the means±SD of 3 independent biological replicates. Letters indicate signifcant variations in values between leaf ranks, as determined using ANOVA followed by a Tukey HSD test (p-value<0.05). [file 13007_2024_1187_MOESM2_ESM.jpg]

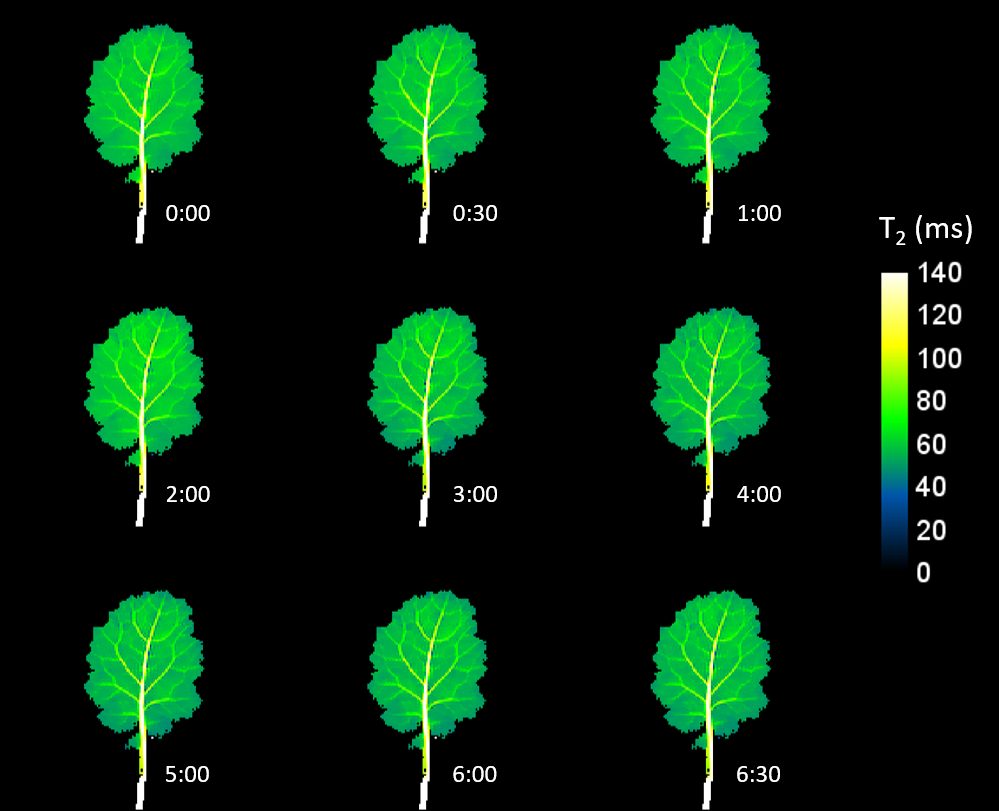

Supplement: Supplementary file 3 — Additional fle 3: Figure S3. Transverse relaxation (T2) maps of a mature leaf (leaf rank 3) over a time period of 6h30min. [file 13007_2024_1187_MOESM3_ESM.jpg]
